# A Novel circRNA hsa_circRNA_002178 as a Diagnostic Marker in Hepatocellular Carcinoma Enhances Cell Proliferation, Invasion, and Tumor Growth by Stabilizing SRSF1 Expression

**DOI:** 10.1155/2022/4184034

**Published:** 2022-08-27

**Authors:** Jing Li, Ting Han, Zhenzhen Li, Hongmei Han, Yingchun Yin, Baohua Zhang, Hengming Zhang, Luan Li

**Affiliations:** ^1^Department of Pathology, Zibo Central Hospital, Zibo 255000, Shandong, China; ^2^Department of Gastroenterology, Qilu Hospital of Shandong University (Qingdao), Qingdao 266035, Shandong, China; ^3^Department of Pediatrics, Zibo Maternal and Child Health Care Hospital, Zibo 255000, Shandong, China; ^4^Department of Pathology, Weifang People's Hospital, Weifang 261000, Shandong, China; ^5^Department of Hepatobiliary Surgery, Qingdao Municipal Hospital of Shandong University, Qingdao 266035, Shandong, China

## Abstract

**Background:**

Previous research studies have shown that the elevation of circular RNA (circRNA), hsa_circRNA_002178, was associated with the poor prognosis of breast cancer and colorectal cancer, while its molecular mechanisms underlying the effects on hepatocellular carcinoma (HCC) are still elusive.

**Methods:**

The microarray dataset GSE97332 was obtained from the Gene Expression Omnibus (GEO) database and calculated by using the GEO2R tool to identify differentially expressed circRNAs. Differentially expressed hsa_circRNA_002178, in 7 HCC tissue samples and paracancerous tissues, as well as in HCC cell lines and normal hepatocytes, was checked by RT-qPCR. Cell proliferation, invasion, migration, and epithelial-to-mesenchymal transition (EMT)-related proteins were tested in hsa_circRNA_002178-overexpressed or hsa_circRNA_002178-knocked down HCC cells. Subsequently, we identified whether hsa_circRNA_002178 binds to serine- and arginine-rich splicing factor 1 (SRSF1) and then analyzed their function in regulating HCC cell behavior. The effect on HCC cell xenograft tumor growth was observed by the knockdown of hsa_circRNA_002178 *in vivo*.

**Results:**

GEO2R-based analysis displayed that hsa_circRNA_002178 was upregulated in HCC tissues. Overexpression or knockdown of hsa_circRNA_002178 encouraged or impeded HCC cell proliferation, migration, invasion, and EMT program. Mechanically, hsa_circRNA_002178 bound to SRSF1 3′-untranslated region (UTR) and stabilized its expression. SRSF1 weakening eliminated the effects of pcDNA-hsa_circRNA_002178 on cell malignant behavior. Finally, the knockdown of hsa_circRNA_002178 was confirmed to prevent xenograft tumor growth.

**Conclusions:**

hsa_circRNA_002178 overexpression encouraged the stability of SRSF1 mRNA expression, and it may serve as an upstream factor of SRSF1 for the diagnosis of HCC.

## 1. Introduction

Primary liver cancer is one of the most common malignancies worldwide, among which hepatocellular carcinoma (HCC) is the most common, accounting for approximately 75–85% [1]. Although scientific and technological advances have facilitated the development of diagnosis and treatment for HCC, surgical techniques including surgery and liver transplantation have improved the prognosis of some patients [2]. However, due to the lack of better biological indicators for early diagnosis, patients tend to present late when liver cancer is detected, and some have even lost surgical opportunity [2]. Moreover, the long-term survival rate of patients with advanced liver cancer is consistently low due to the high rate of metastasis and recurrence of HCC. Therefore, we need to find new biomarkers and therapeutic targets to improve the prognosis of patients with HCC. Most HCCs identified by previous studies are caused by cirrhosis due to viral hepatitis, alcoholism, and metabolic syndrome [3]. Currently, surgical liver transplantation may be the first choice treatment for HCC patients in clinic, but the 5-year survival rate of HCC patients is still unsatisfactory due to poor diagnostic strategies, frequent metastases, lack of effective treatment, and high recurrence rates [4]. However, there are potential mechanisms regarding HCC treatment that have not been clearly elucidated. Recently, many research studies have shown that circular RNAs (circRNAs) are able to participate in HCC tumor generation, suggesting that circRNAs may play a momentous role in HCC prognosis [5].

circRNAs are a kind of noncoding RNAs newly discovered in recent years, which widely exist in organisms. Unlike linear RNAs in general, circRNAs do not have a common 5′cap and 3′ adenosine tail structure. Circular RNAs have been shown to be more stable than linear RNAs [6]. Currently, many circRNAs with differential expression signatures have been identified in HCC tissue samples. For instance, a study displayed that hsa_circRNA_103809 inhibition markedly restrained HCC cell malignant behavior and prevented tumor growth. Additionally, the circRNA_101368 level was ascended in HCC cells, and overexpression of circRNA_101368 is associated with worse prognosis in HCC patients. The interference of circRNA_101368 memorably restrained the cell viability, cytoskeletal generation, and EMT of HCC cells [7]. Furthermore, circRNA_0008274 was found to be highly expressed in HCC, and its high expression enhanced the proliferation, migration, and invasion of HCC cells, while depleting circRNA_0008274 inhibited the malignant biological behaviors of HCC cells [8]. It is a pity that their mechanisms of action in carcinogenesis are not fully understood. Many of the currently known functions of circRNAs include miRNA sponges or RBPs [9]. In this study, based on Gene Expression Omnibus (GEO) data analysis (GSE97332), we unearthed hsa_circRNA_002178 is elevated in HCC. Interestingly, a previous study found that high expression of hsa_circRNA_002178 is associated with poor prognosis in breast cancer. The inhibition of hsa_circRNA_002178 results in reduced cell viability, energy metabolism, and tube formation capacity [10]. Unfortunately, the role and regulatory pathways of hsa_circRNA_002178 in HCC are still unclear. Therefore, we hypothesized hsa_circRNA_002178 may participate in the development of HCC. Subsequently, we performed biological and molecular cellular experiments *in vitro* to examine the effects of hsa_circRNA_002178 and its downstream on the malignant behavior. In this study, we evaluated the expression levels of hsa_circRNA_002178 in HCC patient tissues and cells, and the effects on the malignant cell behavior. The possible potential downstream genes were predicted and validated by the analysis website as well as luciferase reporter gene and RIP assays.

Based on the above reports, this study systematically explored the roles of hsa_circRNA_002178 in the biological functions of HCC cells, aiming to provide useful assistance for HCC clinical diagnosis.

## 2. Materials and Methods

### 2.1. Tissue Sample Collection

A total of 50 cases were selected and included at the Zibo Central Hospital attended patients with HCC. The pathological diagnosis of HCC was carried out according to the standards of the World Health Organization. This study was conducted by Zibo Central Hospital, and ethics approval was obtained by the ethics committee. Inclusion criteria were as follows: the first diagnosis of HCC between 2015 and 2018 (minimum 5 years of potential follow-up), no previous diagnosis of cancer, and no direct evidence of disease within 1 month after the first surgery. All patients underwent routine physical examinations every 3–6 months for the first 5 years of follow-up visits and annually thereafter. Overall survival was defined as the number of months from surgical treatment to death. The seizure-free survival time is defined as the time from surgical treatment to the clinical medical detection of seizures (in months as the enterprise).

### 2.2. Cell Culture

Huh7, Hep3B, HepG2, HCCLM3, SK-hep1, and L02 cell lines were obtained from Shanghai Huiying Biotech Co., Ltd, Shanghai, China. Subsequently, cells were subjected to mycoplasma testing and isoenzyme testing, and cell viability assays were performed by the biological company (GeneCreate Biological Engineering Co., Ltd., Wuhan, China). All cells were cultured in the DMEM containing 10% fetal bovine serum, 100 U/mL penicillin, and 100 *μ*g/mL streptomycin (in which SNU-1 cell culture medium does not contain sodium pyruvate).

### 2.3. Bioinformatics Analysis

Microarray data are available with accession number GSE97332 from Gene Expression Omnibus database (GEO, https://www.ncbi.nlm.nih.gov/geo/). Dataset GSE97332 was based on the platform GPL19978. GSE97332 data included 7 hepatocellular carcinoma primary tumor tissues and 7 adjacent nontumor tissues. Differentially expressed genes between hepatocellular carcinoma primary tumor tissues and paired nontumor tissues were accepted when |log2 fold change| ≥ 1.5 and *p* < 0.01. Gene expression profiling interaction analysis (GEPIA, https://gepia.cancer-pku.cn/) was used to analyze the expression of SRSF1 in hepatocellular tissues and adjacent normal tissues and the 60-month survival rate of HCC patients with high or low expression of hsa_circRNA_002178. Starbase was used to predict the targeting site between hsa_circRNA_002178 and SRSF1 mRNA 3′-untranslated region (UTR).

### 2.4. Genetic Overexpression and Knockdown

The hsa_circRNA_002178 full sequence was ligated into pcDNA3.1 plasmid. The shRNAs were designed by Qiagen to knock down hsa_circRNA_002178 (sh-hsa_circRNA_002178) and SRSF1 (sh-SRSF1). Confluent cells were diluted in the DMEM, and the cells were observed to grow to about 70% confluence when the cell monolayer was covered with the serum-free DMEM. In brief, before transfection, cells were digested with 1% trypsin treatment. After being counted in a blood counting chamber, the cells were plated onto six‐well culture plates for 24 hours and then transfected at 40%–60% confluence. Lipofectamine® 3000 reagent was diluted by using Opti-MEM medium (2 tubes) and mix well sufficiently. DNA master mix was then prepared by diluting the DNA by using Opti-MEM medium, followed by the addition of p3000™ reagent. Lipofectamine® 3000 had been diluted in each tube reagent with diluted DNA (1 : 1 ratio). DNA-liposome complexes were added to the cells after 5-min incubation at room temperature. All cells in each group were collected for subsequent experiments after incubating in an incubator at 37°C with 5% CO_2_ for a specified period of time.

### 2.5. RT-qPCR

Cells from each group were collected, TRIzol (Thermo Fisher, Shanghai, China) was added to extract total cellular RNA, and total cellular RNA was reverse transcribed to cDNA using a reverse transcription kit. Using cDNA as a template, RT-qPCR was performed, and the PCR products were detected with the StepOnePlus real-time PCR system (Thermo Fisher, USA), with three replicate wells set for each group, and GAPDH as the internal reference. The relative expression levels were calculated by using the 2^−ΔΔCT^ method. The sequences of primers were as follows (designed on website https://www.PrimerBank.com): hsa_circRNA_002178 forward, 5′- CTG GTA TCC CTG CAA GTT AAG TC-3′; and reverse, 5′-TGC TCC CGT GGC TGG TCT AAC GCA AA-3′; GAPDH forward, 5′-TGC AGT GGC AAA GTG GAG ATT-3′; and reverse, 5′-TCG CTC CTG GAA GAT GGT GAT-3′.

### 2.6. Western Blotting

Total cell protein was extracted with RIPA lysate, and the protein concentration was determined using a BCA protein assay kit (Thermo Fisher Scientific, Waltham, MA, USA) in a microplate reader. After denaturation for 10 min with the addition of loading buffer, 50 *μ*g protein samples were subjected to SDS-PAGE and transferred onto PVDF membranes. The membrane was blocked with blocking solution (5% nonfat dry milk) for 2 h and subsequently washed three times using TBST. Specific primary and secondary antibodies were next added separately, followed by incubation on a shaker. ImageJ software was applied to detect and analyze the gray values of protein bands on the membrane. The primary antibodies are as follows: rabbit polyclonal anti-*β*-actin antibody (Abcam, ab8227, 1 : 1000), rabbit monoclonal anti-SRSF1 antibody (Abcam, ab133689, 1 : 1000), rabbit monoclonal anti-E-cadherin antibody (Abcam, ab40772, 1 : 10000), and rabbit monoclonal anti-Vimentin antibody (Abcam, ab92547, 1 : 1000).

### 2.7. Cell Viability Assay

The cells were seeded in 96-well culture plates at a cell number per well of 3 × 10^3^, and 5 replicate wells were set 0069 n each group. Transfection was performed after incubation at 37°C in 5% CO_2_ until the cells became adherent. Then, the cells were incubated at 37°C in a 5% CO_2_ incubator for 0, 24, 48, 72, 96, and 120 h after which the supernatant was discarded and 100 *μ*l of complete medium and 10 *μ*L of CCK-8 (Sigma-Aldrich, St. Louis, MO, USA). After incubation at 37°C in 5% CO_2_ for 1 h, the optical density (OD) value at 450 nm was measured using a microplate reader (Bio-Rad, Hercules, CA, USA).

### 2.8. Transwell Invasion Assay

The cell invasion ability was detected by treatment with 8.0-*μ*m chamber plates. The upper surface of the Transwell filter we used was coated with Matrigel (BD, New Jersey, USA). Firstly, cells were planted into the 8.0-*μ*m chamber plates, then 300 *μ*L of serum-free DMEM medium was added to the upper compartment of the chamber, and then, 500 *μ*L of DMEM medium supplemented with 10% FBS was added to the lower chamber for 48-h incubations. Then, the noninvasive cells on the upper side of the chamber were suspended with a cotton swab, and then, the invasive cells were fixed in 4% paraformaldehyde and stained with a crystal violet solution. We stained infiltrating cells by using an Olympus IX70 inverted microscope (Olympus Corp, Tokyo, Japan) and randomly selected the best six fields of view, and each experiment was repeated three times.

### 2.9. Wound-Healing Migration Assay

Cells from each group were seeded in 6-well plates at a concentration of 10^5^ cells/well and then scratched with the tip of a pipette 24 h later. Subsequently, the cell fluid was replaced with a complete medium. Photographs were taken every 24 h. The cells were observed once with an inverted microscope to detect their migration ability.

### 2.10. Luciferase Reporter Assay

Full-length DNA coding sequence of SRSF1 was inserted into pGL3-basic vector, and then, two vectors containing different inserts cloned from hsa_circRNA_002178 were constructed downstream SRSF1. With pGL3-SRSF1-hsa_circRNA_002178 reporter vector transfection of Huh7 cells, roots' luciferase activity of individual groupings was assessed using the dual luciferase reporter system (Thermo Fisher Scientific, Waltham, MA, USA).

### 2.11. RNA Immunoprecipitation (RIP) Assay

Protease inhibitor EDTA as well as RNase inhibitor (Qiagen, Hilden, Germany) was added to IP lysis buffer to lyse cells. After the addition of magnetic beads preclearing for 30 min, IgG antibody and SRSF1 antibody (Abcam, USA) were added, and then, the added magnetic beads were rotated to mix for 2 h at room temperature. Aspirate supernatant on magnetic stand and wash beads with IP lysis buffer. After the removal of proteins by the addition of proteinase K, TRIzol LS was added to extract RNA and finally subjected to subsequent analysis.

### 2.12. RNA Pull-Down Assay

The relationship between hsa_circRNA_002178 and SRSF1 was predicted by Starbase and verified by RNA pull-down assay. Biotinylated hsa_circRNA_002178 was synthesized and transfected into Huh7 cells, and the negative control (NC) was set to demonstrate the specificity of the response. After 48 hours, the cells were washed and collected, and then, the lysate was incubated with avidin-anchored magnetic beads at 4°C for 3 h. After washing the beads sufficiently, the RNA-protein complex was eluted, and the RNA pull-down product was subjected to RT-qPCR.

### 2.13. In Vivo Experiments

Animal experiments were approved and supervised by the Animal Ethics Committee of Shandong University. Five-week-old male athymic BALB/c nude mice were obtained from the Experimental Animal Center of Shandong University (Jinan, China) and subsequently randomly divided into 2 groups, including control and hsa_circRNA_002178 knocking-down. First, we transfected hsa_circRNA_002178 shRNA into Huh7 cells to knock down hsa_circRNA_002178. For the control group, 5 × 10^5^ Huh7 cells were injected subcutaneously into the abdominal cavity of the mice. For the hsa_circRNA_002178 knocking-down group, 5 × 10^5^ Huh7 cells transfected with hsa_circRNA_002178 shRNA were injected subcutaneously into the abdominal cavity of the mice. Tumor length and width were calculated with vernier calipers every 3 days. After 36 days, the mice were humanely sacrificed, and the subcutaneous tumors were excised and removed.

### 2.14. Statistical Analysis

SPSS 22.0 and GraphPad Prism 7.0 were used for data analysis and mapping. The pairwise comparisons were analyzed using the chi-square test. The measurement data were represented as mean ± SEM with normal distribution and homogeneity of variance. Student's *t*-test was performed for the comparison between two groups. The means of the different groups were compared using one-way or two-way analysis of variance (ANOVA) following Tukey's post hoc test. *P* < 0.05 was considered statistically significant. All experiments were repeated 3 times (*n* = 3).

## 3. Results

### 3.1. Hsa_circRNA_002178 Level Was Upregulated in HCC Tissues and Cell Lines

**Figure 1:**
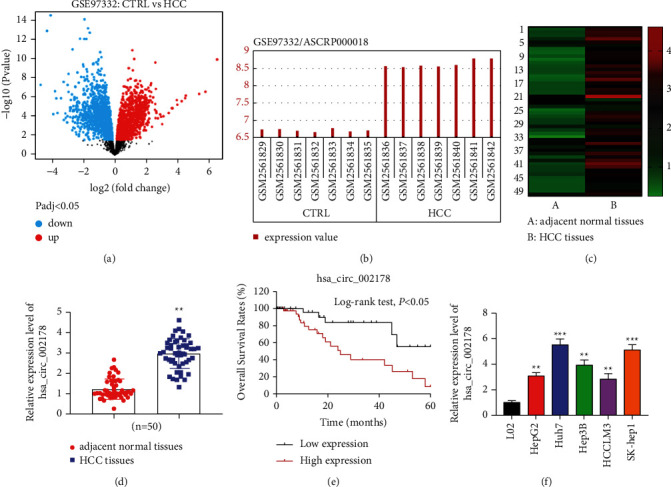
Hsa_circRNA_002178 was increased in HCC. (a) Volcano plot of circRNAs. (b) The hsa_circRNA_002178 expression in 7 HCC samples and 7 normal samples of the GSE83521 dataset. (c) Heatmap of hsa_circRNA_002178 level in 7 HCC and 7 normal samples. (d) The hsa_circRNA_002178 level in HCC and normal tissue samples collected from 50 patients was detected by RT-qPCR, ^*∗∗*^*P* < 0.01 versus adjacent normal tissues. (e) Relative hsa_circRNA_002178 level in HCC cell lines (HepG2, Huh7, Hep3B, SK-hep1, and HCCLM3) and human normal liver cells (L02) was tested by RT-qPCR, ^*∗∗*^*P* < 0.01 and ^*∗∗∗*^*P* < 0.001 versus L02. (f) Overall survival of patients with high hsa_circRNA_002178 level (Red line) or low hsa_circRNA_102415 level (Black line). *N* = 3. *P* < 0.01.

Several studies have elucidated that circRNAs display molecular functions in HCC. Here, the analysis of the HCC dataset GSE97332 downloaded from the GEO database repository revealed that differentially expressed circRNA, hsa_circRNA_002178, was prominently increased in HCC tissues (Figures 1(a)–1(c)). Subsequently, the expression level of hsa_circRNA_002178 was confirmed to be higher in the tumor tissues of HCC patients than in the corresponding normal tissues by PCR results (Figure 1(d)). Similarly, hsa_circRNA_002178 expression was overexpressed in human HCC cell lines, including HepG2, Huh7, Hep3B, HCCLM3, and SK-hep1, compared with human normal hepatocytes (L02), especially in Huh7 and SK-hep1 cells (Figure 1(e)). We then analyzed the overall survival of HCC patients with different hsa_circRNA_002178 levels and discovered that HCC patients with a higher hsa_circRNA_002178 level had significantly lower overall survival (Figure 1(f)).

### 3.2. Overexpression of hsa_circRNA_002178 Facilitated HCC Cell Proliferation, Invasion, and Migration

**Figure 2:**
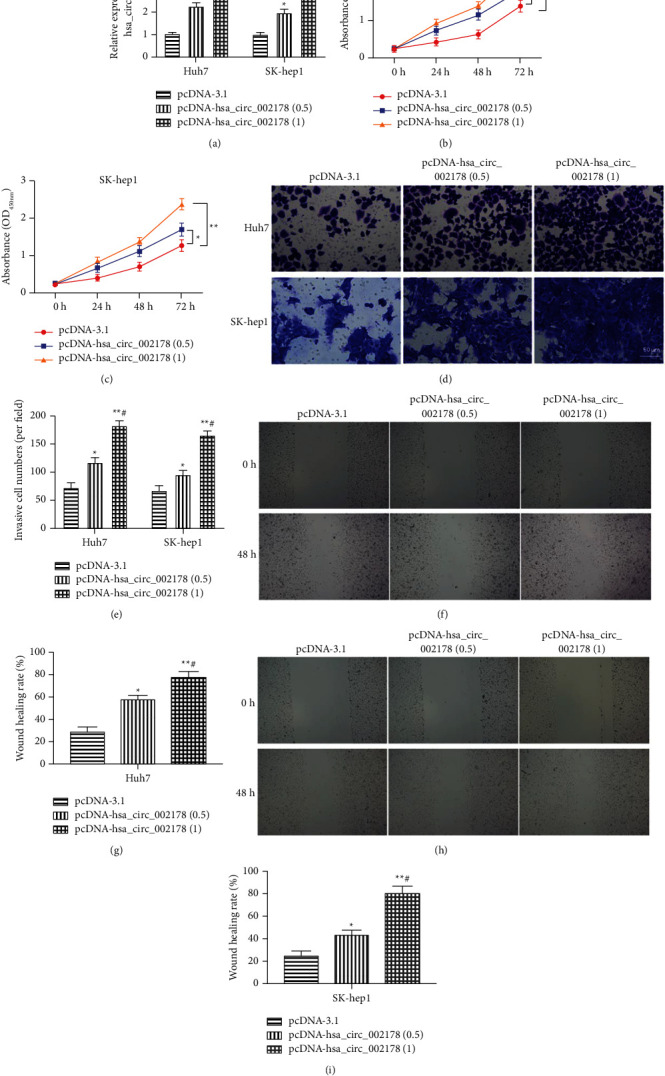
Overexpression of hsa_circRNA_002178 facilitated HCC cell proliferation and invasion. Different doses of pcDNA-hsa_circRNA_002178 were transfected into SK-hep1 and Huh7 cells for 24 h. (a) The transfection efficiency of pcDNA-hsa_circRNA_002178. (b-c) The cell proliferation after transfection with different doses of pcDNA-hsa_circRNA_002178. (d-e)The cell invasion ability of SK-hep1 and Huh7 cells after transfection with different doses of pcDNA-hsa_circRNA_002178. (f-i) The cell migration ability of SK-hep1 and Huh7 cells after transfection with different doses of pcDNA-hsa_circRNA_002178. *N* = 3. *P* < 0.01.

To further explore whether hsa_circRNA_002178 is involved in HCC cell malignant behavior, we established two stable hsa_circRNA_002178-overexpressing cell lines. The overexpression efficiency of pcDNA-hsa_circRNA_002178 in Huh7 and sk-hep1 cells is displayed in Figure 2(a). The CCK-8 results revealed that hsa_circRNA_002178 overexpression observably increased HCC cell proliferation, especially in the high-dose transfection group (Figures 2(b)–2(c), *p* < 0.01). Additionally, we further evaluated the effects of hsa_circRNA_002178 overexpression on the invasion and migration abilities of HCC. As shown in Figures 2(d)–2(i), high hsa_circRNA_002178 expression could substantially increase cell migration and invasion, especially in the high-dose transfection group.

### 3.3. Interference hsa_circRNA_002178 Expression Suppressed Cell Proliferation, Invasion, and Migration

**Figure 3:**
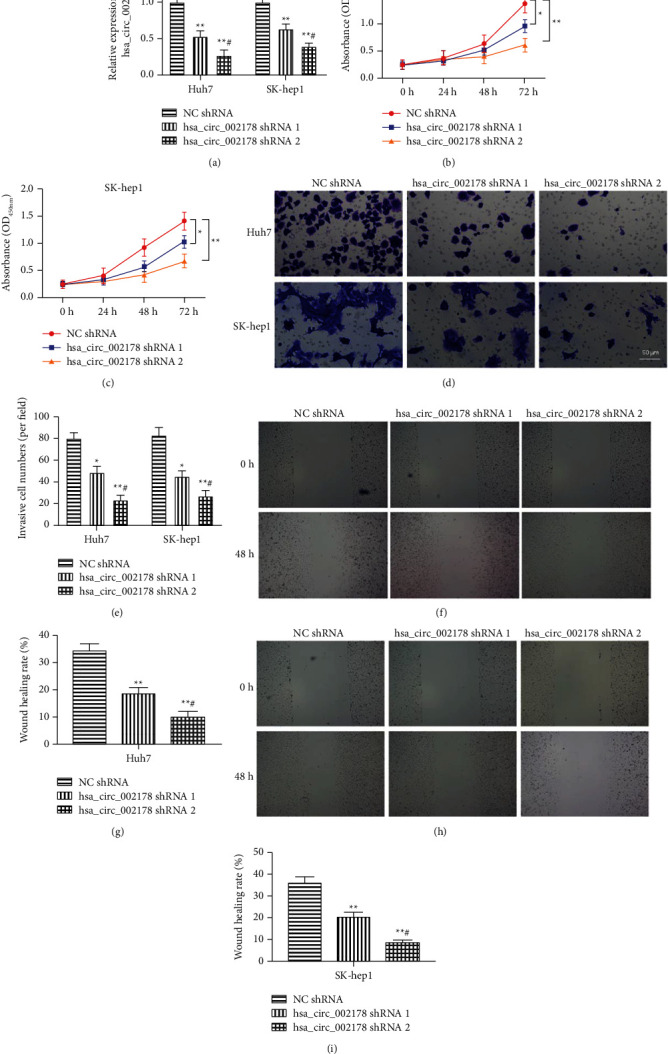
Interference hsa_circRNA_002178 expression inhibited cell proliferation, invasion, and migration. Different doses of hsa_circRNA_002178 shRNA were transfected into SK-hep1 and Huh7 cells for 24 h. (a) The transfection efficiency of hsa_circRNA_002178 shRNA in SK-hep1 and Huh7 cells was tested. (b–c) The cell proliferation after transfection with different doses of hsa_circRNA_002178 shRNA was employed by CCK-8. (d-i) The cell invasion and migration abilities of SK-hep1 and Huh7 cells after transfection with different doses of hsa_circRNA_002178 shRNA was employed by Transwell and damage repair assays. *N* = 3. *p* < 0.01.

Subsequently, SK-hep1 and Huh7 cells were transfected with 1 *μ*g or 2 *μ*g hsa_circRNA_002178 shRNA and its negative control for 24 h, respectively. And the resulting cell lines exhibited memorably reduced the hsa_circRNA_002178 level in a dose-dependent manner (Figure 3(a), *p* < 0.01). The CCK-8 assay displayed that hsa_circRNA_002178 knockdown inhibited SK-hep1 and Huh7 cell proliferation (Figures 3(b)–3(c), *p* < 0.01). Furthermore, we evaluated the effect of hsa_circRNA_002178 interference on cell invasion and migration. hsa_circRNA_002178 interference markedly inhibited the cell invasion and migration, in which the high-dose group of hsa_circRNA_002178 shRNA had prominently higher inhibitory effects on cell proliferation and invasion its low-dose transfection group (Figures 3(d)–3(i), *p* < 0.05).

### 3.4. hsa_circRNA_002178 Could Positively Regulate SRSF1 Expression

**Figure 4:**
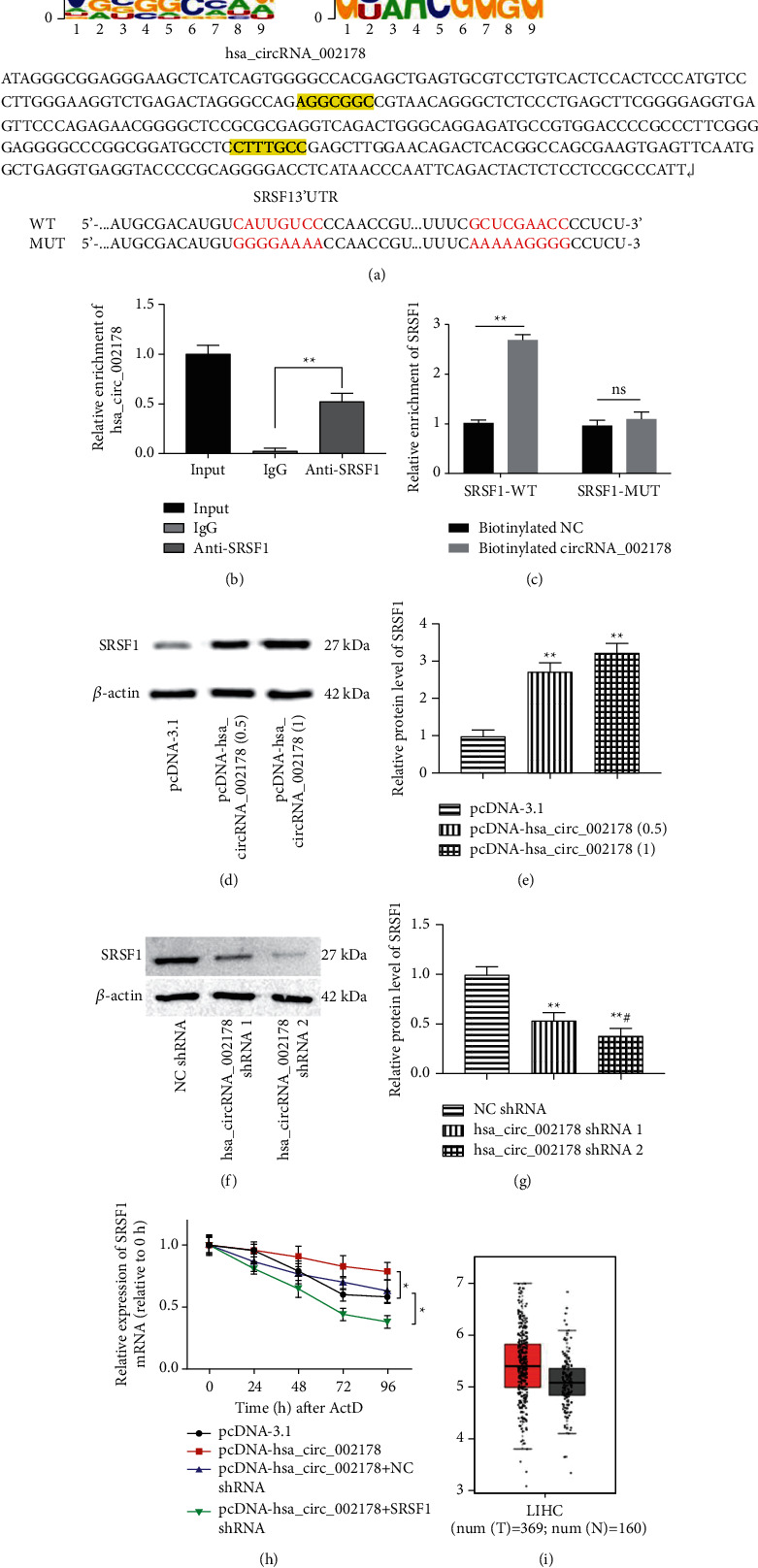
hsa_circRNA_002178 positively regulated SRSF1 expression. (a) The SRSF1 motifs and their possible targets on hsa_circRNA_002178. (b) RIP assay was used to assess the binding relationship of hsa_circRNA_002178 and SRSF1. (c) RNA pull-down assay was performed to further verify the combination of hsa_circRNA_002178 and SRSF1. (d-e) The protein level of SRSF1 in Huh7 cells after transfection with different doses of pcDNA-hsa_circRNA_002178. (f-g) The protein expression of SRSF1 in Huh7 cells after transfection with different doses of hsa_circRNA_002178 shRNA was employed by Western blotting. (h) The rates of the degradation of SRSF1 mRNA in cells were detected by RT-qPCR. (i)The expression of SRSF1 in HCC was predicted by bioinformatics website analysis. *N* = 3. *P* < 0.01.

Next, we used the online website (https://jaspar.genereg.net/) to predict targets that may be regulated by hsa_circRNA_002178. Figure A displays the SRSF1 motifs and its binding sites to hsa_circRNA_002178. To further verify whether hsa_circRNA_002178 could directly target with SRSF1, the RNA pull-down and RIP assays were performed. The RIP assay has displayed that a higher hsa_circRNA_002178 level was detected in anti-SRSF1immuno-precipitates relative to control IgG immune precipitates (Figure 3(b), *p* < 0.01). Then, the RNA pull-down results are shown in Figure 4(c), SRSF1 expression in the biotinylated hsa_circRNA_002178 group was prominently upregulated compared to the NC group. To further verify the regulatory relationship, pcDNA-hsa_circRNA_002178 and sh-hsa_circRNA_002178 vectors were transfected into Huh7 cells. hsa_circRNA_002178 overexpression markedly elevated the SRSF1 protein level, whereas hsa_circRNA_002178 silencing suppressed SRSF1 expression in Huh7 cells (Figures 3(d)–3(g), *p* < 0.01). Moreover, we discovered that hsa_circRNA_002178 overexpression could increase the SRSF1 mRNA level after treating with actinomycin *D* (ActD, a transcriptional inhibitor), while hsa_circRNA_002178 inhibition showed the opposite result. This finding revealed that SRSF1 mRNA stability was added in hsa_circRNA_002178-upregulated cells and declined in hsa_circRNA_002178-depleted cells (Figure 4(h), *p* < 0.05). Subsequently, we found that the SRSF1 level was significantly higher in HCC tissues than in normal tissues (Figure 4(i)).

### 3.5. Knockdown of SRSF1 Antagonized the Promotive Effects of hsa_circRNA_002178 Upregulation on HCC Cell Functions

**Figure 5:**
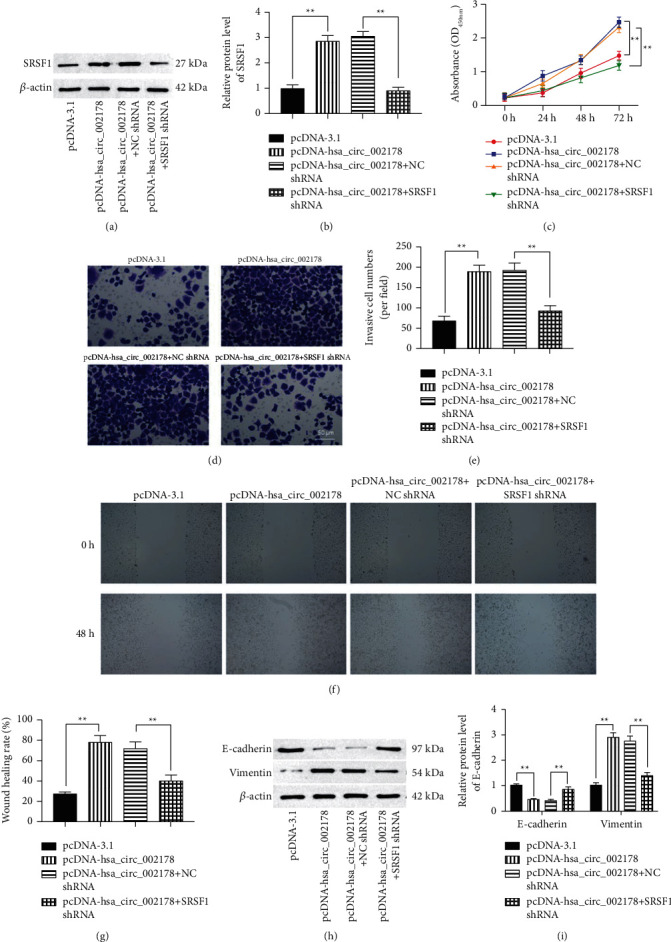
Knockdown of SRSF1 antagonized the promotive effects of hsa_circRNA_002178 upregulation on HCC cell proliferation, invasion, and migration. pcDNA-hsa_circRNA_002178 was transfected alone or together with SRSF1 shRNA into Huh7 cells. (a-b) The efficiency of SRSF1 shRNA in Huh7 cells was tested by Western blotting. (c) The cell proliferation was tested by CCK-8 assay. (d-g)The cell invasion and migration abilities of Huh7 cells after transfection with pcDNA-hsa_circRNA_002178 or/and SRSF1 shRNA were detected by Transwell or damage repair assays. (h-i) The E-cadherin and Vimentin protein levels in Huh7 cells after transfection with pcDNA-hsa_circRNA_002178 or/and SRSF1 shRNA were employed by Western blotting. *N* = 3. *p* < 0.01.

Next, we further evaluated whether hsa_circRNA_002178 and SRSF1 are functionally associated, and rescue experiments were performed by co-transfectingpcDNA-hsa_circRNA_002178 with SRSF1 shRNA plasmids. Figure A and B display the transfection efficiency as SRSF1 shRNA. From the cellular behavior, knockdown of SRSF1 prominently reversed the promoting of hsa_circRNA_002178 upregulation on the Huh7 cell functions, which was reflected by cell proliferation, invasion, and migration (Figures 5(c)–5(g), *p* < 0.05). Additionally, the effects of hsa_circRNA_002178 on cell proliferation and invasion implied that EMT may mediate the roles of hsa_circRNA_002178 in HCC. The results displayed that hsa_circRNA_002178 overexpression reduced the E-cadherin level and raised the Vimentin level, suggesting that hsa_circRNA_002178 overexpression induced EMT of HCC cells, which were memorably reversed by SRSF1 inhibition (Figures 5(h)–5(i), *p* < 0.01).

### 3.6. SRSF1 Overexpression Reversed the Effects of hsa_circRNA_002178 Inhibition on HCC Cell Malignant Behaviors

**Figure 6:**
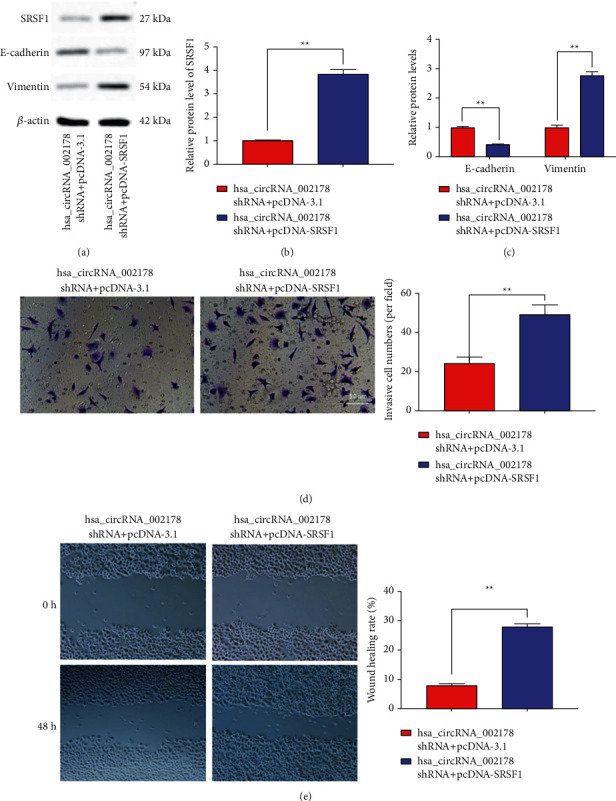
SRSF1 overexpression reversed the effects of hsa_circRNA_002178 inhibition on HCC cell malignant behaviors. hsa_circRNA_002178 shRNA was transfected alone or together with pcDNA- SRSF1 into Huh7 cells for 24 h. (a-c) Western blotting illustrated the SRSF1, E-cadherin, and Vimentin expression in Huh7 cells transfection with hsa_circRNA_002178 shRNA or/and pcDNA-SRSF1. (d) Transwell assay illustrated the invasion in Huh7 cells transfection with hsa_circRNA_002178 shRNA or/and pcDNA-SRSF1.  (e) The damage repair assay illustrated the migration in Huh7 cells transfection with hsa_circRNA_002178 shRNA or/and pcDNA-SRSF1. *N* = 3. *p* < 0.01.

Subsequently, hsa_circRNA_002178 shRNA was transfected alone or together with pcDNA-SRSF1 into Huh7 cells. The efficiency of pcDNA-SRSF1 on its expression level in Huh7 cells is shown in Figures 6(a) and 6(b). SRSF1 overexpression prominently downregulated E-cadherin protein levels (Figures 6(a) and 6(c), *p* < 0.01) in cells subjected to hsa_circRNA_002178 interference, as well as memorably increased vimentin level (Figures 6(a) and 6(c), *p* < 0.01), cell invasion (Figure 6(d), *p* < 0.01), and cell migration (Figure 6(e), *p* < 0.01). In summary, SRSF1 upregulation could reverse the effects of hsa_circRNA_002178 restrain on HCC cell EMT program, invasion, and migration.

### 3.7. Interference of hsa_circRNA_002178 Suppressed HCC Cell Growth In Vivo

**Figure 7:**
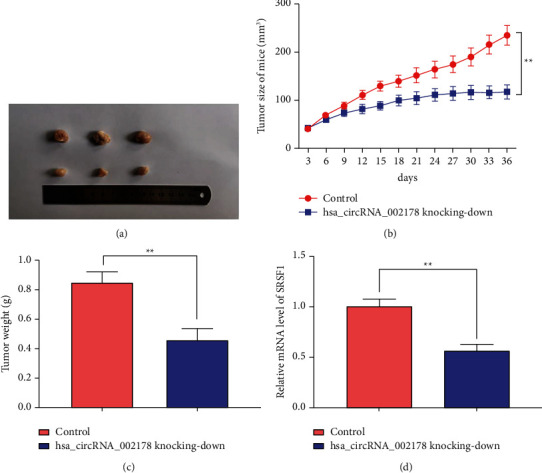
Knockdown of hsa_circRNA_002178 suppressed HCC cell growth in vivo. hsa_circRNA_002178 shRNA plasmids were transfected into Huh7 cells, and the cells were subsequently injected subcutaneously into the abdomen of nude mice. (a) Representative images of tumors. (b) Tumor volume. (c) Tumor weight. (d) The SRSF1 mRNA level in mouse HCC tissues was checked by using Western blotting. *N* = 3. *p* < 0.01.

To confirm our in vitro results that hsa_circRNA_002178 inhibition could restrain HCC growth, the effects of hsa_circRNA_002178 knockdown in a xenograft murine model were next evaluated. Nude mice were subcutaneously injected with Huh7 cells stably transfected with hsa_circRNA_002178 shRNA plasmid to further evaluate the effect of hsa_circRNA_002178 on HCC cell growth in vivo. The tumor weight and volume were prominently decreased in the hsa_circRNA_002178 knockdown group (Figures 7(a)–7(c), *p* < 0.01). Furthermore, compared to the control group, the SRSF1 mRNA level was prominently decreased in the hsa_circRNA_002178 knocking-down group, which was consistent with our previous *in vitro* findings. In conclusion, the knockdown of hsa_circRNA_002178 functions as a tumor suppressor by restraining tumor growth *in vivo* and *in vitro*.

## 4. Discussion

The survival of HCC patients has improved after current clinical medical treatments, but the overall survival time of HCC patients is still poor [11]. In addition, to date, only a few markers have been discovered that can play a hallmark role in the identification and prognosis of liver cancer [12]. Recent research studies have shown that circRNAs may be associated with HCC initiation and progression, mainly through the dysregulation of circRNAs in HCC tumor tissues [13,14]. circRNA CDRlas plays a contributory role in HCC disease development, and its expression was significantly higher in HCC tissues than in adjacent normal tissues. Not only that, the expression amount of circRNA CDRlas was considered to be significantly correlated with hepatic microvascular infiltration [15]. A research has revealed that circRNA-MAST1 was ascended in HCC, the inhibition of circRNA-MAST1 could restrain HCC development [16]. Moreover, circADAMTS14 might suppress HCC cell viability via regulating the downstream target gene levels [17]. In the present study, our study displayed that the hsa_circRNA_002178 level was rose in HCC. It has been reported that hsa_circRNA_002178 was upregulated in breast cancer cells and tissues and regulates breast cancer biological functions, such as proliferation, migration, invasion, and apoptosis [18]. Besides, Wang et al. study found that the strong expression of hsa_circRNA_002178 was detected in exosomes from the plasma of lung adenocarcinoma patients and may serve as a biomarker for the early diagnosis of lung adenocarcinoma [19]. Data from Yang et al. displayed that the expression of hsa_circRNA_002178 was significantly higher in oral squamous cell carcinoma tissue specimens than in paracancerous tissues. In addition, patients with high expression of hsa_circRNA_002178 had a higher advanced pathological stage and incidence of distant metastasis, and a lower overall survival rate [20]. hsa_circRNA_002178 was substantially increased in colorectal cancer clinical tissue samples, and its upregulation could encourage colorectal cancer cell invasion, proliferation, colony formation, and glycolysis, thus driving colorectal cancer development. Here, by performing CCK-8, damage repair, and Transwell assays, our findings demonstrated that the interference of hsa_circRNA_002178 restrained HCC cell proliferation, migration, and invasion abilities. In recent years, several possible functions of circRNAs have been suggested, such as regulation of gene transcription and protein turnover through the binding of circRNAs to microRNAs or proteins [9, 21, 22].

A large number of studies have shown that circular RNAs have significant tissue specificity in different tissues and can function as endogenous competitors of mRNA to exert biological effects [23]. These lines of evidence all suggest that circRNAs have important biological roles in life processes [24–26]. circRNAs have been found to be able to compete with other molecules with shared RBP binding motifs for binding to specific RBPs [27]. The circRHOBTB3 could bind to HuR, which is a ubiquitously expressed and functional RBP in colorectal cancer, and promote colorectal cancer cell malignant behaviors [28]. Knockdown of circRNA hsa_circ_0062270 suppressed melanoma tumor growth *in vivo* by inhibiting RBP EIF4A3 expression [29]. Furthermore, CircSMARCA5 could regulate angiogenesis in glioblastoma multiforme through the binding of SRSF1 [30]. In our study, depleting hsa_circRNA_002178, which is upregulated in HCC cells, caused decreased SRSF1, indicating that SRSF1 might be a regulatory target for hsa_circRNA_002178 in HCC cells.

Serine-/arginine-rich splicing factor 1 (SRSF1) is a representative member of the alternative splicing factor family, which exerts the function of alternative splicing by recognizing and binding to corresponding splice sites [31]. SRSF1 is mainly involved in the regulation of body homeostasis, intracellular localization, trafficking of mRNA, processing of miRNA, mediating mRNA nonsense degradation, and the regulation of cell cycle and apoptosis [32, 33]. SRSF1 has been reported to regulate tumorigenesis through multiple pathways. SRSF1 exerts oncogenic roles in breast cancer partially by regulating the alternative splicing of PTPMT1, which could be a therapeutic target candidate in breast cancer and a prognostic factor in HR + breast cancer patients [34]. Emerging evidence has found that SRSF1 expression is significantly increased in HCC patients prevented [35]. SRSF1 could accelerate the malignant behavior of HCC [36]. Furthermore, a study confirmed that low LINC02580 expression was associated with poor prognosis in HCC patients, and LINC02580 regulated epithelial mesenchymal transition (EMT)-related pathways in HCC cells by specifically binding to SRSF1 [37]. According to GEPIA data, the SRSF1 level in HCC tissues was higher than that in normal tissues, and the knockout of SRSF1 weakened the increase of proliferation and migration. Further SRSF1 rescue experiments showed that SRSF1 gene knockout blocked the suppressed effect of hsa_circRNA_002178 overexpression on the proliferation, migration, and invasion of HCC cells.

Our findings demonstrated that hsa_circRNA_002178 knockdown inhibited the malignant behavior of HCC cells. Besides, the effect of hsa_circRNA_002178 interference was proved in the HCC mouse xenograft model. With the development of the theory of precision medicine, the diagnosis and treatment of HCC have risen to the molecular level, and the expression and mutation detection of related genes have gradually become the basis of their clinical treatment. The present study identified the roles of hsa_circRNA_002178 in HCC cells, which may provide potential therapeutic targets and schemes for HCC treatment.

## Data Availability

The datasets used during the present study are available from the corresponding author on reasonable request.

## Abbreviations:

HCC: Hepatocellular carcinoma
GEO: Gene expression omnibus
(circRNA): Circular RNA
(SRSF1): Serine- and arginine-rich splicing factor 1
(EMT): Epithelial-to-mesenchymal transition
(UTR): 3′-Untranslated region.
